# Identification and Validation of Potential Ferroptosis-Related Genes in Glucocorticoid-Induced Osteonecrosis of the Femoral Head

**DOI:** 10.3390/medicina59020297

**Published:** 2023-02-06

**Authors:** Ning Chen, Yuan Meng, Huixian Zhan, Gang Li

**Affiliations:** 1 Postdoctoral Research Center, Shandong University of Traditional Chinese Medicine, Jinan 250355, China; 2 College of Clinical Medicine, Guangzhou University of Chinese Medicine, Guangzhou 510006, China; 3 Orthopaedic Microsurgery, Affiliated Hospital of Shandong University of Traditional Chinese Medicine, Jinan 250014, China; 4 The First Clinical Medical School, Shandong University of Traditional Chinese Medicine, Jinan 250355, China

**Keywords:** glucocorticoid-induced osteonecrosis of the femoral head, ferroptosis, bioinformatics analysis, gene expression omnibus

## Abstract

*Background and Objectives.* Glucocorticoid-induced osteonecrosis of the femoral head (GIONFH) is a serve complication of long-term administration of glucocorticoids. Previous experimental studies have shown that ferroptosis might be involved in the pathological process of GIONFH. The purpose of this study is to identify the ferroptosis-related genes and pathways of GIONFH by bioinformatics to further illustrate the mechanism of ferroptosis in SONFH through bioinformatics analysis. *Materials and Methods*. The GSE123568 mRNA expression profile dataset, including 30 GIONFH samples and 10 non-GIONFH samples, was downloaded from the Gene Expression Omnibus (GEO) database. Ferroptosis-related genes were obtained from the FerrDb database. First, differentially expressed genes (DEGs) were identified between the serum samples from GIONFH cases and those from controls. Ferroptosis-related DEGs were obtained from the intersection of ferroptosis-related genes and DEGs. Only ferroptosis DEGs were used for all analyses. Then, we conducted a Kyoto encyclopedia of genome (KEGG) and gene ontology (GO) pathway enrichment analysis. We constructed a protein–protein interaction (PPI) network to screen out hub genes. Additionally, the expression levels of the hub genes were validated in an independent dataset GSE10311. *Results*. A total of 27 ferroptosis-related DEGs were obtained between the peripheral blood samples of GIONFH cases and non-GIONFH controls. Then, GO, and KEGG pathway enrichment analysis revealed that ferroptosis-related DEGs were mainly enriched in the regulation of the apoptotic process, oxidation-reduction process, and cell redox homeostasis, as well as HIF-1, TNF, FoxO signaling pathways, and osteoclast differentiation. Eight hub genes, including TLR4, PTGS2, SNCA, MAPK1, CYBB, SLC2A1, TXNIP, and MAP3K5, were identified by PPI network analysis. The expression levels of TLR4, TXNIP and MAP3K5 were further validated in the dataset GSE10311. *Conclusion*. A total of 27 ferroptosis-related DEGs involved in GIONFH were identified via bioinformatics analysis. TLR4, TXNIP, and MAP3K5 might serve as potential biomarkers and drug targets for GIONFH.

## 1. Introduction

Glucocorticoid-induced osteonecrosis of the femoral head (GIONFH) is a pathologic process of bone cell death that commonly affects young and middle-aged adults [1]. GIONFHprimarily manifests clinically as hip pain in the early stages, followed by the collapse of the femoral head, eventually leading to dysfunction of the hip joint [2]. The occurrence of GIONFH has been rising over the past decades due to the extensive application of glucocorticoids for viral infections, spinal trauma, and autoimmune and hematopoietic diseases [3]. Moreover, glucocorticoid therapy is now recommended for a large number of patients with severe COVID-19 [4]. It has been reported that osteonecrosis develops in 9–40% of patients receiving long-term glucocorticoid therapy [5]. The occurrence and development of GIONFH is a complex processwith multiple steps and genes. It is a widespread consensus that the repair process of osteonecrosis requires precisely coordinated bone resorptive functions of osteoclasts and bone-forming functions of osteoblasts [6]. The precise pathogenesis and molecular mechanisms underlying GIONFHhave not yet been fully elucidated, and there is currently a lack of effective clinical treatments. Therefore, it is important to investigate the molecular mechanism underlying GIONFH.

Ferroptosis is emerging as a novel type of programmed cell death discovered in recent years [7]. Ferroptosis is characterized by reactive oxygen species (ROS) accumulation, iron overload, and lipid peroxidation, ultimately leading to oxidative cell death [8].Iron overload, an initiator of ferroptosis, could increase bone resorption and decrease bone formation [9]. Moreover, iron overload could increase the number of osteoclasts [10]. The morphological characteristics of ferroptosis are decreased mitochondrial size, increased mitochondrial membrane density, and reduced mitochondrial crista and mitochondrial outer-membrane rupture [11]. Major efforts in terms of ferroptosis have been focused on the field of cancer. Although the role of iron in osteonecrosis has not yet been reported until now, recent evidence indicates that ferroptosis is closely related to the pathogenesis of GIONFH. An iron overload could promote osteoclast differentiation and bone resorption by producing ROS [12] and inhibiting osteogenic differentiation of mesenchymal stem cells [13]. Meanwhile, dexamethasone (Dex), an artificially synthetic glucocorticoid, could induce ferroptosis via P53/SLC7A11/GPX4 pathway in GIONFH [14].However, manyferroptosis-related genes involved in GIONFH have not yetbeen found, so further studies of ferroptosis-related genesinvolved in GIONFH need to be explored.

To date, there have been no bioinformatics-based studies on the pathogenesis of ferroptosis-related GIONFH. In the present study, we analyzed the expression of ferroptosis-related differentially expressed genes (DEGs) between the serum samples of GIONFH patients and controls based on the microarray dataset. Then, the Gene Ontology (GO) and Kyoto Encyclopediaof Genes and Genomes (KEGG) databases were usedfor the enrichment analysis of ferroptosis-related DEGs to establish the pathogenesis of GIONFH. Moreover, we constructed a protein–protein interaction(PPI) network to identify hub genes based on ferroptosis-related DEGs. This study may improve the understanding of ferroptosis-related mechanisms underlying GIONFH.

## 2. Materials and Methods

### 2.1. Data Information

The mRNA expression profile dataset of GSE123568 was downloaded from the Gene Expression Omnibus (GEO) database (https://www.ncbi.nlm.nih.gov/geo/, accessed on 1 October 2022), which was based on the platform of GPL15207 Affymetrix Human Gene Expression Array. This dataset contains 30 serum samples from GIONFH patients and 10 serum samples from non-GIONFH controls. Among them, GIONFH patients were aged (23.07 ± 3.01) years, while non-GIONFH controls were aged (25.02 ± 2.87) years.

### 2.2. Ferroptosis-Related Genes Detection

FerrDb (http://www.zhounan.org/ferrdb/current/, accessed on 1 October 2022) is the first manually curated database for ferroptosis covering regulatory factors and molecular markers for ferroptosis and ferroptosis-related diseases worldwide. In total, 649 ferroptosis-related genes were collected from the FerrDb database (driver, n = 255; marker, n = 125; suppressor, n = 208) and a literature search (n = 61). After eliminating duplicate items from the above whole genes, we identified 397 ferroptosis-related genes.

### 2.3. Ferroptosis-Related DEGs Analysis

The linear model for microarray data (LIMMA) package was used to identify DEGs between the serum samples from GIONFH cases and those from controls. Adjusted *p*-value < 0.05 and |log_2_ fold change (FC)| ≥1 were used as the selection criteria. Ferroptosis-related DEGs were obtained from the intersection of ferroptosis-related genes and DEGs. The pheatmap package was used to draw a heatmap to show ferroptosis-related DEGs in R software.

### 2.4. GO and KEGG Enrichment Analyses of Ferroptosis-Related DEGs

DAVID Bioinformatics Resources 6.8 (https://david.ncifcrf.gov/, accessed on 1 October 2022) was an online tool comprehending the biological function of the ferroptosis-related DEGs in this study. Moreover, KEGG enrichment analysis was applied to annotate the pathways of ferroptosis-related DEGs. A *p*-value of <0.05 and Gene counts of ≥3 were set as the cutoff criteria for significant enrichment. The results of enrichment analyses were visualized using ggplot2 and GOplot packages.

### 2.5. PPI Network Analysis

The Search Tool for the Retrieval of Interacting Genes (STRING) database (https://string-db.org/, accessed on 1 October 2022) was an open accessible resource to demonstrate interactions between proteins encoded by DEGs. In the present study, the PPI network of ferroptosis-related DEGs was constructed via STRING with the default threshold of a combined score >0.4. Finally, visualization of the PPI network was obtained with Cytoscape software (version 3.9.1). The CytoHubba plugin was adopted to calculate the degree of all ferroptosis-related DEGs. Only ferroptosis-related DEGs with a degree of ≥10 were identified as hub genes.

### 2.6. Validation of the Hub Genes

To validate the reliability of the dataset GSE123568, the expression levels of hub genes were further checked in an independent dataset GSE10311. Dataset of GSE10311, including three samples of human primary osteoblast-like cells treated with Dex for 24 h (GSM260305, GSM260306 and GSM260307) and three untreated samples of human primary osteoblast-like cells (GSM260323, GSM260324 and GSM260325).

## 3. Results

### 3.1. Identification of Ferroptosis-Related DEGs

**Figure 1:**
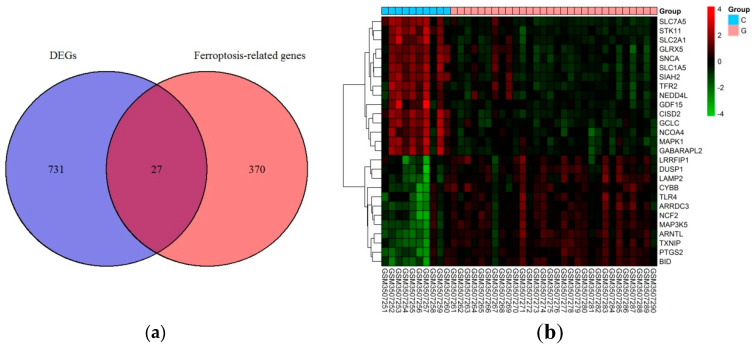
Ferroptosis-related differentially expressed genes in GIONFH and non-GIONFH samples. (**a**) Venn diagram of ferroptosis-related differentially expressed genes after the intersection between differentially expressed genes and ferroptosis-related genes. (**b**) Heatmap of the 27 ferroptosis-related differentially expressed genes in GIONFH and non-GIONFH samples.

**Figure 2:**
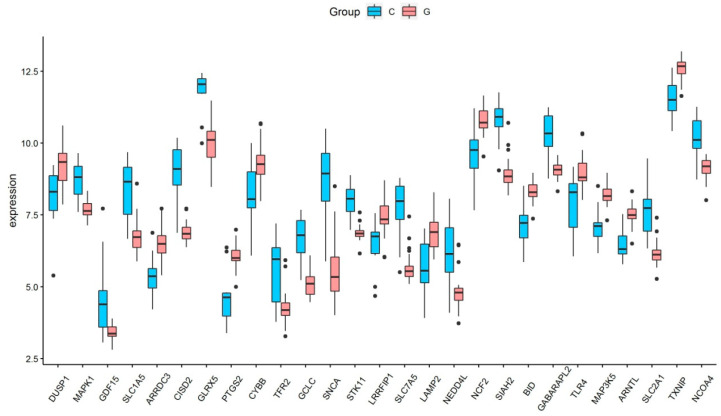
The expression levels of 27 ferroptosis-related differentially expressed genes in GIONFH and non-GIONFH samples.

Based on the selection criteria, an aggregate of 758 DEGs was acquired in the dataset of GSE123568, of which 457 were up-regulated, and 301 were down-regulated. After the intersection with 397 ferroptosis-related genes, a total of 27 ferroptosis-related DEGs were identified (Figure 1a). These ferroptosis-related DEGs were shown in the heatmap (Figure 1b). Of these ferroptosis-related DEGs, 12 were up-regulated, and 15 were down-regulated. The expression patterns of ferroptosis-related DEGs are summarized in Figure 2.

### 3.2. GO and KEGG Enrichment Analyses of Ferroptosis-Related DEGs

**Figure 3:**
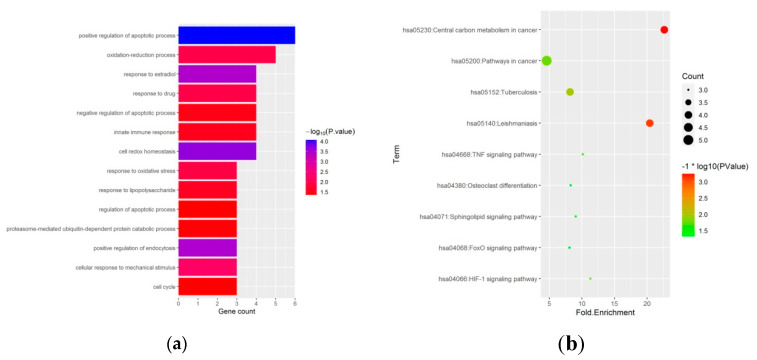
GO, and KEGG functional enrichment analysis of 27 ferroptosis-related differentially expressed genes in GIONFH and non-GIONFH samples. (**a**) GO functional enrichment analysis of 27 ferroptosis-related differentially expressed genes in GIONFH and non-GIONFH samples. (**b**) KEGG pathway analysis of 27 ferroptosis-related differentially expressed genes in GIONFH and non-GIONFH samples.

To reveal the biological functions of the ferroptosis-related DEGs, we conducted GO function and KEGG enrichment analysis by DAVID. GO enrichment analysis revealed that ferroptosis-related DEGs were mainly enriched in the regulation of the apoptotic process, oxidation-reduction process, and cell redox homeostasis (Figure 3a and Table 1). Moreover, KEGG enrichment analysis showed that the ferroptosis-related DEGs were mainly enriched in the HIF-1 signaling pathway, TNF signaling pathway, osteoclast differentiation, and FoxO signaling pathway (Figure 3b and Table 2).

### 3.3. PPI Network Analysis

**Figure 4:**
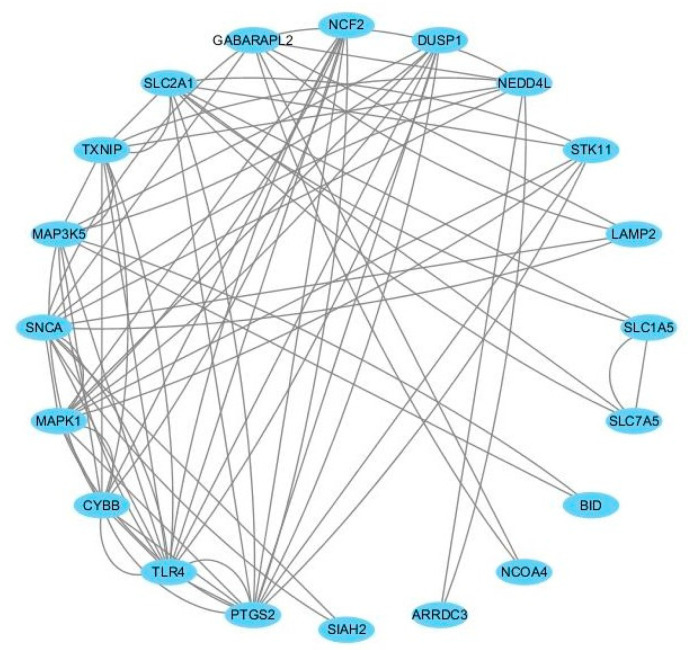
The protein–protein interaction network of the 8 hub genes in GIONFH and non-GIONFH samples.

To further reveal the potential interactions among ferroptosis-related DEGs, a PPI network was constructed using the STRING database. After removing the isolated and partially connected nodes, a total of 21 nodes and 37 edges in the network were visualized by the Cyctoscape, as shown in Figure 4. In the PPI network, eight hub genes with the highest node degrees were identified as follows: TLR4, PTGS2, SNCA, MAPK1, CYBB, SLC2A1, TXNIP, and MAP3K5. Among them, TLR4, PTGS2, CYBB, TXNIP, and MAP3K5 were significantly up-regulated, while SNCA, MAPK1, and SLC2A1 were significantly down-regulated compared with healthy controls.

### 3.4. Validation of the Hub Genes

**Figure 5:**
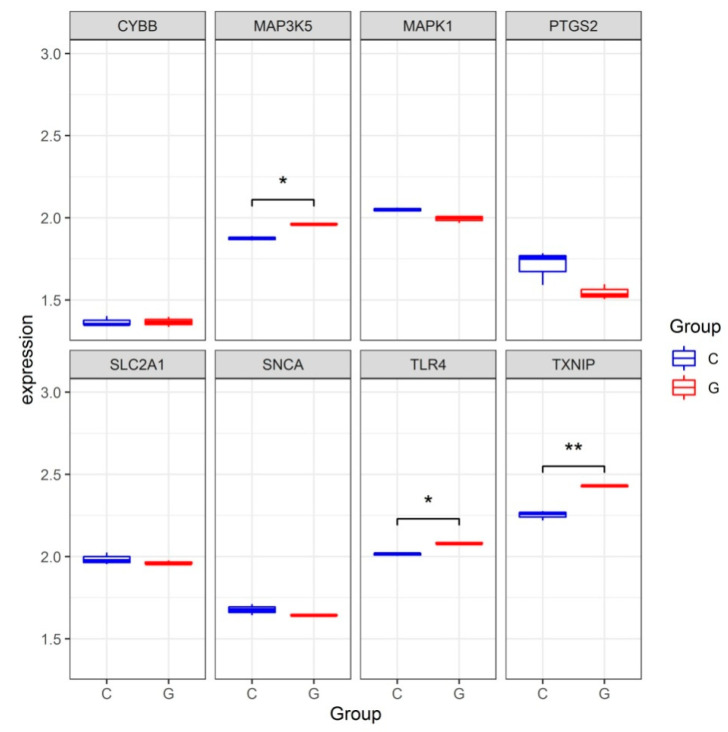
The expression levels of hub genes in the gene expression dataset GSE13011. * *p* < 0.05, ** *p* < 0.01.

To validate the reliability of the dataset GSE123568, the expression levels of hub genes were further identified in the dataset GSE10311. Consistent with the results in the dataset GSE123568, the expression levels of TLR4, TXNIP and MAP3K5 were significantly increased in the dataset GSE10311. The expression level of CYBB was increased but without significance. The expression levels of SNCA, MAPK1 and SLC2A1 were all decreased but without significance (Figure 5).

## 4. Discussion

GIONFH is a metabolic disease caused by the high-dose administration of glucocorticoids, leading tonecrosis ofbone cells and the bone marrow. It is a challenging disorder characterized by femoral head collapse and subsequent dysfunction of the hip joint, which severely decreases the quality of life. It is estimated that 20,000 to 30,000 new patientsare diagnosed with osteonecrosis in the United States [15], and 100,000 new cases annuallyinSouth Korea [16]. The incidence of NONFH is high in young and middle-aged adults. Its morbidity has been rising partly due to the increasing prevalence of many associated diseases and/or risk factors, as well as the raised awareness of this condition. Total hip arthroplasty (THA)is currently the most widely used surgical procedure for ONFH treatment [17]; however, in young patients with indications for THA, the possibility of frequent revisions at short intervals and future revisions could be challenging [18]. GIONFH is one of the world’s public health problems that have attracted the widespread attention of society. In spite of many pathophysiological mechanisms for GIONFH being proposed, such as vascular insufficiency, osteocyte apoptosis, bone fragility, and fat embolism, the exact pathogenic mechanism remains unknown. The term iron ptosis, created in 2012, describes a form of regulatory cell death induced by small molecules of erastin, which inhibits the cysteine-glutamate reverse transporter and leads to the consumption of glutathione. In recent years, ferroptosis has gained a great deal of attention as iron-related regulated cell death. Accumulating evidence indicates that ferroptosis plays an important role in the pathogenesis of GIONFH. Experimental data on the cell model of GIONFH demonstrated that Dex could induce ferroptosis [14]. The involvement of iron metabolism in bone growth and lipid peroxidation in femoral head osteonecrosis suggests that ferroptosis is involved in this pathogenesis of GIONFH [19]. However, the exact mechanism of ferroptosis in bone homeostasis regulation remains largely unknown, and it is yet unclear whether ferroptosis is a driver or a passenger event in bone homeostasis. Understanding the underlying mechanisms of ferroptosis in GIONFH may give detailed insights into the pathogenesis of GIONFH.

Microarray analysis using high-throughput platforms is a promising and efficient tool for the investigation of the molecular mechanisms of disease. To the best of our knowledge, the present study, for the first time, exploredferroptosis-related genes and pathways in GIONFH via bioinformatics analysis. In this study, we identified 27 ferroptosis-related DEGs associated with GIONFH based on the intersection of DEGs from the serumexpression profile dataset of GIONFH compared with controls. Of these 27 genes, 12 were up-regulated, and 15 were down-regulated. Someof these genes involved in GIONFH have been previously studied. For example, Lin et al. [20] demonstrated that MAPK1 was down-regulated in blood samples of GIONFH patientscompared with healthy controls. Li et al. [21] found that TLR4 was elevated in serum samples of GIONFH patientscompared with non-GIONFHs. Chen et al. [22] indicated that the level of PTGS2 was increasingly up-regulated with the progression of this disease. The above literature reports support the evidence that these candidate genes identified here may be associated with the onset and progression of GIONFH.

The integrity of bone is maintained through a precise dynamic balance between osteogenic and osteoclastic activities, and the bone regeneration process is a continuous cycle [23]. Osteoblasts and osteoclasts are mainly involved in bone reconstruction, including the formation, mineralization, construction and resorptionof bone [24]. The ultimate aim of establishing a dynamic balance between the two cell types is to replace old/damaged bone with new/healthy bone [25]. Therefore, strategies to develop effective therapies for ONFH may be directed at activating osteoblasts or inhibiting osteoclasts. In contrast to patients with OA, patients with ONFH had abnormal osteoblast replication capacity in the proximal femur [26]. Research has demonstrated the crucial role of ferroptosis in regulating bone homeostasis. Osteoporosis is a bone metabolic diseasein which osteoclast-mediated bone resorption exceeds osteoblast-mediated bone formation. Osteoclasts, osteoblasts, osteocytes, and stem cells are also affected bythe regulation of ferroptosis in osteoporosis. Ferroptosis-related genes may contribute to the onset and/or progression of GIONFH by regulating bone homeostasis.

To clarify the underlying molecular mechanisms of these ferroptosis-related DEGs, we evaluated their biological functionsthrough GO and KEGG enrichment analysis. GO results showed that the ferroptosis-related DEGs were significantly enriched inpositive regulation of the apoptotic process, oxidation-reduction process, and cell redox homeostasis. Apoptosis is aphysiological process of cell death that has a noteworthy role during the development and growth, and maintaining the skeleton [27]. At the molecular level of bone cells, glucocorticoids regulate various functions, including the apoptotic process [28]. Moreover, Dex can induce apoptosis of primary osteoblasts [29]. It has been proven that GIONFH is closely associated with the apoptosis of osteocytes and osteoblasts [30]. Weinstein et al. [31] reported a 3-fold increase in the apoptosis rate of osteoblasts in mice treated with glucocorticoids for 4 weeks, while the apoptosis rate of osteoblasts and osteocytes was also higher in patients with glucocorticoid-induced osteoporosis. They suggested that increased osteoblast and/or osteoclast apoptosis in the femoral head may be one of the main features of the pathogenesis of GIONFH [26]. Ferroptosis is characterized by the production of ROS and lipid peroxidation. ROS are natural byproducts of aerobic metabolism and are produced by all living multicellular organisms. ROS are produced by healthy cells in a highly regulated fashion in order to maintain intracellular redox homeostasis [32]. Oxidative stress can be described as an imbalance between the production of oxidant species and the antioxidant defenses, which may affect cellular redox homeostasis leading to molecular alterations and thus resulting in cell damage [33]. In addition, several recent studies have shown that the overproduction of ROS, disruption of the antioxidant stress system and production of oxidative DNA are important mechanisms of osteoblast apoptosis and osteoclastogenesis [34,35]. Oxidative stress-induced apoptosis can increase lipid peroxidation and decrease the differentiation of bone marrow mesenchymal stem cells into osteoblasts, thereby decreasing bone mass and reducing bone formation [36]. We suspected that there is a crosstalk between ferroptosis and apoptosis regulated by glucocorticoids in GIONFH. Currently, available studies cannot fully reveal the relationship between ferroptosis and apoptosis, and more research is needed in this area. Previous studies reported that Dex induces the imbalance of redox homeostasis [37]. Furthermore, ferroptosis isdriven by lethal lipid peroxidation, a consequence of cellular metabolism and imbalanced redox homeostasis [38]. An important component of the defense against ROS is the capacity of the cell to buffer the redox environment. KEGG results showed that the ferroptosis-related DEGs were mainly enriched in osteoclast differentiation, as well as HIF-1, TNF, and FoxO signaling pathways. HIF-1αis a master regulator of cellular response to hypoxia. Xu et al. [39] demonstrated that over-expressed HIF-1α resisted Dex-induced apoptosis in a hypoxic environment. Implantation of bone marrow mesenchymal stem cellsoverexpressing HIF-1α into femoral heads of GIONFH mice significantly reduced osteonecrotic areas and enhanced bone repair [40]. Sun et al. [41] found that Dex induces osteoblast apoptosis via theFoxO signaling pathway. Therefore, regulating the above signaling pathways may be one of the promising methods to treat GIONFH.

Thereafter, the PPI network of ferroptosis-related DEGs was constructed by using the STRING database and Cytoscape. After removing the isolated and partially connected nodes, a total of 21 nodes and 37 edges were involved in the network. A total of 8 hub genes were screened out, including TLR4, PTGS2, SNCA, MAPK1, CYBB, SLC2A1, TXNIP, and MAP3K5. The expression levels of TLR4, TXNIP and MAP3K5 were validated in an independent dataset GSE10311. The expression levels of TLR4, TXNIP and MAP3K5 were all higher compared to controls in both datasets, GSE123568 and GSE10311. TLR4 is one of the most common members in the Toll-like receptors family, and it is also the most thoroughly studied. TLR4 is mainly expressed in immune cells, namely, T cells, natural killer cells, dendritic cells, macrophages, and neutrophils [42]. It has been demonstrated that the important regulated role of TLR4 in the proliferation and differentiation of osteoclasts [43]. Tian et al. [44] found thatmethylprednisolone, a kind of glucocorticoid, could induce osteonecrosis of the femoral head in rats via the activation of a TLR4 signaling pathway in osteoclast. In addition, a bioinformatic study found that TLR4 was screened out as one of the hub genes enriched in the ferroptosis pathway in child sepsis [45]. Extracellular TLR4 contains a ligand-bound leucine repeat sequence domain that recognizes a variety of ligands, such as lipopolysaccharideand heat shock proteins 60 and 70 [46]. TLR4 triggers the activation of the inflammatory pathway. Inhibition of TLR4 inhibited the expression of ferroptosis-related proteins and decreased the expression of ferroptosis-related genes in hypoxic-ischemic brain damage [47]. The above literature suggests that inhibition of the expression of TLR4 may be a potential drug target for GIONFH treatment. TXNIP is responsible for the regulation of cellular reactive oxygen species. It has been reported that TXNIP causes cellular oxidative stress and inflammationin diabetes [48]. Mo et al. [49] have revealed that the expression levels ofTXNIP were elevated in the serum and bone of a rat model ofglucocorticoid-induced osteoporosis. Another study by Lekva T et al. [50] has proffered that TXNIP knockdown in osteoblasts boosts cell differentiationand strengthens alkaline phosphatase activity. The level of TXNIP was also demonstrated to be highly up-regulated in diabetic retinopathy [48]. TheTXNIP upregulation may cause mitochondrial dysfunction, mitophagy, and ferritinophagy [51]. MAP3K5, also known as apoptosis signal-regulating kinase 1, is a member of the p38 and Jun N-terminal kinase (JNK) mitogen-activated protein kinase (MAPK) pathway, which playsan important role inoxidative stress [52]. Consistent with the high expression level of MAP3K5 in the present study, MAP3K5 was highly expressed in rectal cancer compared to healthy tissues [53]. MAP3K5 was also identified as one of the ferroptosis-related genes in sepsis by bioinformatics analysis [54]. The above literature suggests that inhibition of the expression ofMAP3K5may be a potential drug target for GIONFH treatment. SLC2A1, a facilitative glucose transporter, is responsible for constitutive or basal glucose uptake [55]. Wu et al. [56] have suggested that SLC2A1 may be related to the occurrence of GIONFH. In addition to the genesthat have already been discussed, there is a lack of dataregarding whetherMAP3K5, CYBB, and SNCAhave strongconnections with GIONFH occurrence and progression.

Although potential ferroptosis-related genes involved in GIONFH were identified based on bioinformatics, there were still some limitations of this study. First, the number of samples included in the present study was limited. The use of additional datasets with increased sample sizes in future studies would increase the accuracy and reliability of identified DEGs. Second, further experiments in vitro and in vivo are still needed to validate the ferroptosis-related DEGs and their potential mechanisms.

## 5. Conclusions

A total of 27 ferroptosis-related DEGs involved in GIONFH were identified via bioinformatics analysis. TLR4, TXNIP, and MAP3K5 might serve as potential biomarkers and drug targets forGIONFH.

## Figures and Tables

**Table 1:** GO functional enrichment analysis of 27 ferroptosis-related differentially expressed genes in GIONFH and non-GIONFH samples.

GO Terms	Count	Enriched Genes	*p*-Value
positive regulation of apoptotic process	6	TXNIP, SNCA, PTGS2, DUSP1, MAP3K5, BID	8.51 × 10^−5^
oxidation-reduction process	5	SNCA, PTGS2, NCF2, GLRX5, CYBB	1.23 × 10^−2^
cell redox homeostasis	4	GCLC, NCF2, GLRX5, CYBB	2.23 × 10^−4^
response to estradiol	4	TXNIP, PTGS2, DUSP1, BID	3.66 × 10^−4^
response to drug	4	TXNIP, SNCA, PTGS2, CYBB	1.12 × 10^−2^
innate immune response	4	TLR4, NCF2, MAP3K5, CYBB	2.80 × 10^−2^
negative regulation of apoptotic process	4	SNCA, GCLC, DUSP1, SIAH2	3.23 × 10^−2^
positive regulation of endocytosis	3	SNCA, TFR2, NEDD4L	3.47 × 10^−4^
cellular response to mechanical stimulus	3	TLR4, PTGS2, GCLC	5.36 × 10^−3^
response to oxidative stress	3	PTGS2, GCLC, DUSP1	1.24 × 10^−2^
response to lipopolysaccharide	3	TLR4, SNCA, PTGS2	2.64 × 10^−2^
proteasome-mediated ubiquitin-dependent protein catabolic process	3	SIAH2, NEDD4L, ARNTL	3.90 × 10^−2^
regulation of apoptotic process	3	DUSP1, BID, GDF15	4.26 × 10^−2^
cell cycle	3	TXNIP, SIAH2, MAPK1	4.40 × 10^−2^

**Table 2:** KEGG pathway analysis of 27 ferroptosis-related differentially expressed genes in GIONFH and non-GIONFH samples.

KEGG Terms	Count	Enriched Genes	*p*-Value
hsa05200: Pathways in cancer	5	PTGS2, NCOA4, MAPK1, SLC2A1, BID	1.69 × 10^−2^
hsa05230: Central carbon metabolism in cancer	4	SLC7A5, MAPK1, SLC2A1, SLC1A5	5.67 × 10^−4^
hsa05140: Leishmaniasis	4	TLR4, PTGS2, NCF2, MAPK1	7.69 × 10^−4^
hsa05152: Tuberculosis	4	TLR4, MAPK1, BID, LAMP2	1.03 × 10^−2^
hsa04066: HIF-1 signaling pathway	3	TLR4, MAPK1, SLC2A1	2.55 × 10^−2^
hsa04668: TNF signaling pathway	3	PTGS2, MAP3K5, MAPK1	3.12 × 10^−2^
hsa04071: Sphingolipid signaling pathway	3	MAP3K5, MAPK1, BID	3.85 × 10^−2^
hsa04380: Osteoclast differentiation	3	NCF2, MAPK1, CYBB	4.51 × 10^−2^

## Data Availability

Publicly available datasets were analyzed in this study. These datasets can be found at the following websites: FerrDb (http://www.zhounan.org/ferrdb/current/, accessed on 1 October 2022), GSE123568 (https://www.ncbi.nlm.nih.gov/geo/query/acc.cgi?acc=GSE123568, accessed on 1 October 2022) and GSE10311 (https://www.ncbi.nlm.nih.gov/geo/query/acc.cgi?acc=GSE10311, accessed on 1 October 2022).
